# Age-dependent decrease in dental pulp cavity volume as a feature for age assessment: a comparative in vitro study using 9.4-T UTE-MRI and CBCT 3D imaging

**DOI:** 10.1007/s00414-021-02603-1

**Published:** 2021-04-26

**Authors:** Maximilian Timme, Jens Borkert, Nina Nagelmann, Adam Streeter, André Karch, Andreas Schmeling

**Affiliations:** 1 Institute of Legal Medicine, University Hospital Münster, Röntgenstraße 23, 48149 Münster, Germany; 2 Translational Research Imaging Center (TRIC), Department of Clinical Radiology, University Hospital Münster, Albert-Schweitzer-Campus 1, 48149 Münster, Germany; 3 Institute of Epidemiology and Social Medicine, University of Münster, Domagkstraße 3, 48149 Münster, Germany

**Keywords:** Age estimation, Secondary dentin formation, Magnetic resonance imaging, Cone beam CT, Dental age

## Abstract

Evaluation of secondary dentin formation is generally suitable for age assessment. We investigated the potential of modern magnetic resonance imaging (MRI) technology to visualize the dental pulp in direct comparison with cone beam computed tomography (CBCT). To this end, we examined 32 extracted human teeth (teeth 11–48 [FDI]) using 9.4-T ultrashort echo time (UTE)-MRI and CBCT (methods). 3D reconstruction was performed via both manual and semi-automatic segmentation (settings) for both methods in two runs by one examiner. Nine teeth were also examined by a second examiner. We evaluated the agreement between examiners, scan methods, and settings. CBCT was able to determine the pulp volume for all teeth. This was not possible for two teeth on MRI due to MRI artifacts. The mean pulp volume estimated by CBCT was consistently higher (~ 43%) with greater variability. With lower variability in its measurements, evaluation of pulp volume using the MRI method exhibited greater sensitivity to differences between settings (*p* = 0.016) and between examiners (*p* = 0.009). The interactions of single-rooted teeth and multi-rooted teeth and method or setting were not found to be significant. For examiner agreement, the mean pulp volumes were similar with overlapping measurements (ICC > 0.995). Suitable for use in age assessment is 9.4-T UTE-MRI with good reliability and lower variation than CBCT. For MRI, manual segmentation is necessary due to a more detailed representation of the interior of the pulp cavity. Since determination of pulp volume is expected to be systematically larger using CBCT, method-specific reference values are indispensable for practical age assessment procedures. The results should be verified under in vivo conditions in the future.

## Introduction

Doubtful age information or a complete lack of information about a person’s age could pose challenges to states under the rule of law. In these cases, forensic age estimation can contribute to the implementation of transparent rule of law procedures. Efforts focus on the application of age-appropriate rule-of-law measures. For example, children should be provided with age-appropriate procedures and interventions to prevent harm to them [1–6].

Forensic age assessment then applies evidence-based procedures, in which the chronological age of a person is assessed, based on the developmental status of certain feature systems. In accordance with the recommendations of the Study Group on Forensic Age Diagnostics, both tooth development and skeletal development are included in the examinations [7]. Once the development of all feature systems from skeleton and dentition is complete, degenerative features must generally be used to assess age [8–11]. Secondary dentin formation by odontoblasts in the pulp cavity of teeth has long been reported as one such degenerative feature [9, 12, 13]. Secondary dentin formation can also be assessed non-invasively, as it is associated with the volume reduction of the pulp cavity. Secondary dentin can therefore be used as a feature for age determination in the living and in the deceased [14].

Complications can arise from the fact that diseases can influence the known tooth characteristics and, in particular, the decrease in dental pulp cavity volume [15, 16]. Thus, teeth can also form new dentin as a general defense reaction [17–19]. This so-called tertiary dentin cannot be radiologically distinguished from secondary dentin. Tertiary dentin is formed by odontoblasts, where odontoblasts detect pathogens due to their location at the dentin-pulp junction and their cellular processes in the dentine tubules before the pathogens reach the pulp [20]. Odontoblasts detect specific molecules (pathogen-associated molecular patterns (PAMPs)) and initiate the tooth’s defense response [20]. In the case of minor tooth damage (e.g., exposed dentin areas in caries, abrasion, attrition, and erosion processes), these PAMPs can penetrate the dentin via damage to the enamel. Tertiary dentin is also produced in the case of severe stimuli (e.g., rapidly progressing caries; direct pulp capping; large, exposed dentin areas due to massive loss of tooth structure), which lead to the destruction of the odontoblasts and to the differentiation of hard substance-forming cells (also called odontoblast-like cells) from undifferentiated ecto-mesenchymal cells. Growth factors seem to play a decisive role in this process [21]. Some authors distinguish tertiary dentin into reaction and repair dentin for these reasons [20, 21]. Since the formation of tertiary dentin is not directly associated with chronological age, it has to be avoided that this is incorrectly considered as secondary dentin. For this reason, it is essential that pathological teeth with caries, restorations, severe wear, or apical foci are not included in age assessment.

Recently, attempts have been made to develop practically applicable methods to assess secondary dentin formation in a reproducible manner for age assessment [10, 22–24]. In addition, corresponding reference values were presented, with which to compare the extent of secondary dentin formation present in each case [8, 25, 26]. For this purpose, staging for the assessment of secondary dentin formation in two-dimensional (2D) X-ray such as orthopantomogram (OPTG) or periapical radiography has already been described [10, 24, 25, 27]. However, there are contradictory statements on the applicability of certain methods in OPTG in the literature, which altogether shows a particular shortcoming of the method [22, 25, 28, 29]. Other approaches also examined the 2D area of the dental pulp, but in representative sectional planes from three-dimensional (3D) cone beam computed tomography (CBCT) datasets [30]. These approaches are able to support the basic suitability of secondary dentin formation for age estimation, but must be reviewed critically overall, since secondary dentin formation is a 3D process that cannot easily be captured through conventional 2D X-ray imaging. Consequently, the use of three-dimensional dental imaging to assess the size of the pulp chamber was investigated by various working groups. Based on current data, 3D-CBCT appears to be well suited for assessing secondary dentin formation in living individuals [31–34]. Fundamentally, however, some questions remain unanswered. On the one hand, it is the subject of current research into which tooth is best suited for the assessment of secondary dentin formation [35]. In addition, it has not been conclusively clarified whether the evaluation of the root pulp yields any additional benefit for the accuracy of the age assessment.

Volume assessment is based on the condition that the imaging data have been reconstructed into 3D models. A major hurdle is the accurate identification of anatomical structures within the volumes [36]. This requires that the structures to be examined have been correctly segmented in the imaging beforehand [37]. Thus, segmentation is a factor that must be taken into account as soon as one starts to deal with the analysis of 3D datasets [38]. Selection of the “correct” segmentation technique for a given application has already been outlined as a difficult task [38]. The procedure of different methods for image segmentation has also been described in detail using CBCT datasets for dental age assessment by dental pulp volume [39]. For MRI technology, the best technique for segmenting datasets to visualize the dental pulp is still unclear. Moreover, segmentation methods must always be reassessed in the light of new technical developments.

In 2020, Timme et al. demonstrated that 9.4-T ultrashort echo time (UTE)-magnetic resonance imaging (MRI) technology is capable of imaging the pulp with such accuracy that the datasets could be used for age assessment [40]. It was also highlighted that the specific characteristics of the UTE sequences in particular are suitable for visualizing the tooth structures. Able to image both crown pulp and root pulp is 9.4-T UTE-MRI in both single- and multi-rooted teeth with high spatial resolution, comparable to the accuracy that can be achieved with CBCT [40].

The aim of the present study was to compare 9.4-T UTE-MRI in imaging the dental pulp with the current technology, CBCT. In addition, the aim was to verify whether semi-automatic segmentation yields different results compared to manual segmentation of the datasets.

## Materials and methods

The study was evaluated and approved by the responsible ethics committee (2017–215-f-S). All participants signed a consent form for the use of their teeth for scientific purpose.

For the present study, a total of 32 extracted human teeth were examined by MRI and CBCT. The tooth set of a physiological dentition with teeth 11–48 (according to FDI scheme) was compiled.

The teeth came from eight males and 5 females aged 18–78 years. All teeth were extracted for medical indication. Only teeth that had no visible lesions on inspection immediately before extraction were included in the study. The teeth were rinsed under water and placed in 70% ethanol immediately after extraction.

In preparation for MRI scanning, the tooth was embedded in 1% agarose in a falcon tube and stored at 4 °C overnight. In this configuration, the teeth were also placed in the CBCT after the MRI examination, but without prior refrigeration.

### Materials

MRI was performed on a 9.4-T Bruker Biospec 94/20 (Bruker BioSpin GmbH, Ettlingen, Germany) equipped with a 35-mm quadrature birdcage coil (Rapid Biomedical, Rimpar, Germany). The falcon tube with the embedded tooth was well positioned directly into the middle of the 35-mm micro-coil and fixed. A 3D UTE sequence was performed with the following parameters: time to repetition, 8.0 ms; time to echo, 0.020 ms; flip angle, 5°; averages, 4; scan time, 1 h 12 min; number of projections, 134,526; polar undersampling, 1.52; matrix, 256^3^. Due to the different types of teeth examined, the field of view and spatial resolution had to be adjusted for each tooth.

CBCT was performed on an Orthophos SL Scanner (Dentsply Sirona Inc., York, PA, USA). The sample tube was positioned centrally on the chin rest of the scanner. The scanner was operated with the following parameters: voltage, 85 kV; amperage, 6 mA; FoV, 8 × 8 × 8 cm^3^; 943 mGycm^2^; rotation angle, 204°; scan time, 14.2 s; reconstructed voxel edge length, 8 × 8 HD 160 µm.

### Settings

3D reconstruction of imaging datasets was analyzed with AMIRA software (Version 5.4, Thermo Fisher Scientific Inc., Waltham, MA, USA).

Manual segmentation was performed in such a way that the observer assigned the structures to either the pulp or the tooth structure in each plane in the transversal and sagittal sectional views. Consequently, in each slice, the anatomical structures were marked separately by the examiner.

The semi-automatic segmentation was performed with the so-called magic wand tool of AMIRA software. The “magic wand tool” bases selection on the voxel color gradient. Thus, looking at the raw MRI data, the structures are classified according to their MRI intensity. The automatically selected intensity range was adjusted manually to the structures. The range was adjusted until the region of the selection visually matched the area of the anatomic structure exactly. However, this adjustment of the assignment was only carried out in one slice. For segmentation of the other slices then, there was no further manual correction of the selection made by the tool, even in the case where the segmentation in other slices obviously did not correspond to the anatomical structures.

Thus, all examined teeth were measured by both methods: MRI and CBCT. The two datasets of each tooth were then segmented using both settings: manually and semi-automatically.

In addition, all teeth were segmented a second time by the examiner using both settings to determine the repeatability within a single examiner.

Furthermore, the datasets of 9 randomly selected teeth were segmented again by a second independent examiner using both methods and settings to determine agreement between examiners (reproducibility).

### Statistics

Agreement between examiners, scan methods, and settings was based on the intraclass correlations (ICC) obtained from the decomposition of one-way analysis of variance (ANOVA) models, following the methods of Shrout and Fleiss (1979), and of McGraw and Wong (1996) [41, 42]. The items being rated were the teeth and absolute agreement was analyzed, one at a time, for each study characteristic (examiner, scan method, and scan setting). Potential modifying effects of key attributes on each other were also investigated by fitting their interaction term in a multilevel model with teeth as a random effect to account for correlation between measurements on the same tooth. Pulp data were skewed, necessitating a logarithmic transformation to support linear modelling. Inference was made at the 5% significance level.

## Results

**Fig. 1:**
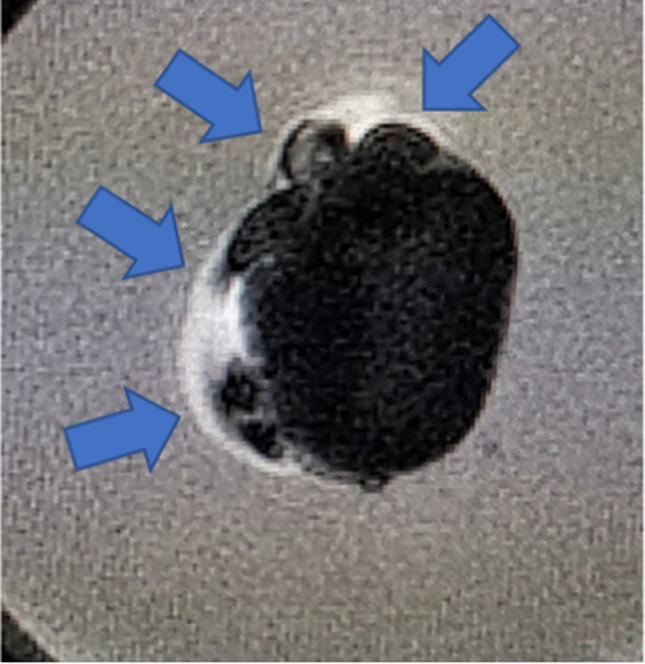
Tooth 31, transversal view, MRI dataset. Susceptibility of artifacts at the junction of the tooth and embedding material (blue arrows)

All datasets from CBCT were analyzable and could be processed into 3D models. For MRI, two teeth (teeth 15 and 31) could not be evaluated. This was likely due to susceptibility artifacts as signal dropouts and image distortion (Fig. 1) from contamination arising from the in vitro nature of this study, namely air ingress into the interior of the sample or blood residues on the outer tooth.

In addition, caries was detected in two teeth (teeth 12 and 47). These were not detected during the clinical inspection, but subsequently by both MRI and CBCT methods, and confirmed by probing the teeth. Although unsuitable for practical age assessment, the teeth were left in the study for the present work.

The calculated volumes of the pulp ranged from 2.92 (tooth 32) to 96.50 mm^3^ (tooth 18) for the CBCT examinations. For the MRI examinations, the values for the volume of the pulp were between 0.92 (tooth 22) and 85.66 mm^3^ (tooth 38). It has to be taken into account that all third molars are from an 18-year-old male. When the third molars are excluded from consideration, the largest pulp volumes were 64.42 mm^3^ (tooth 46) and 35.79 mm^3^ (tooth 27) according to CBCT and MRI, respectively.

### Examiner agreement

**Fig. 2:**
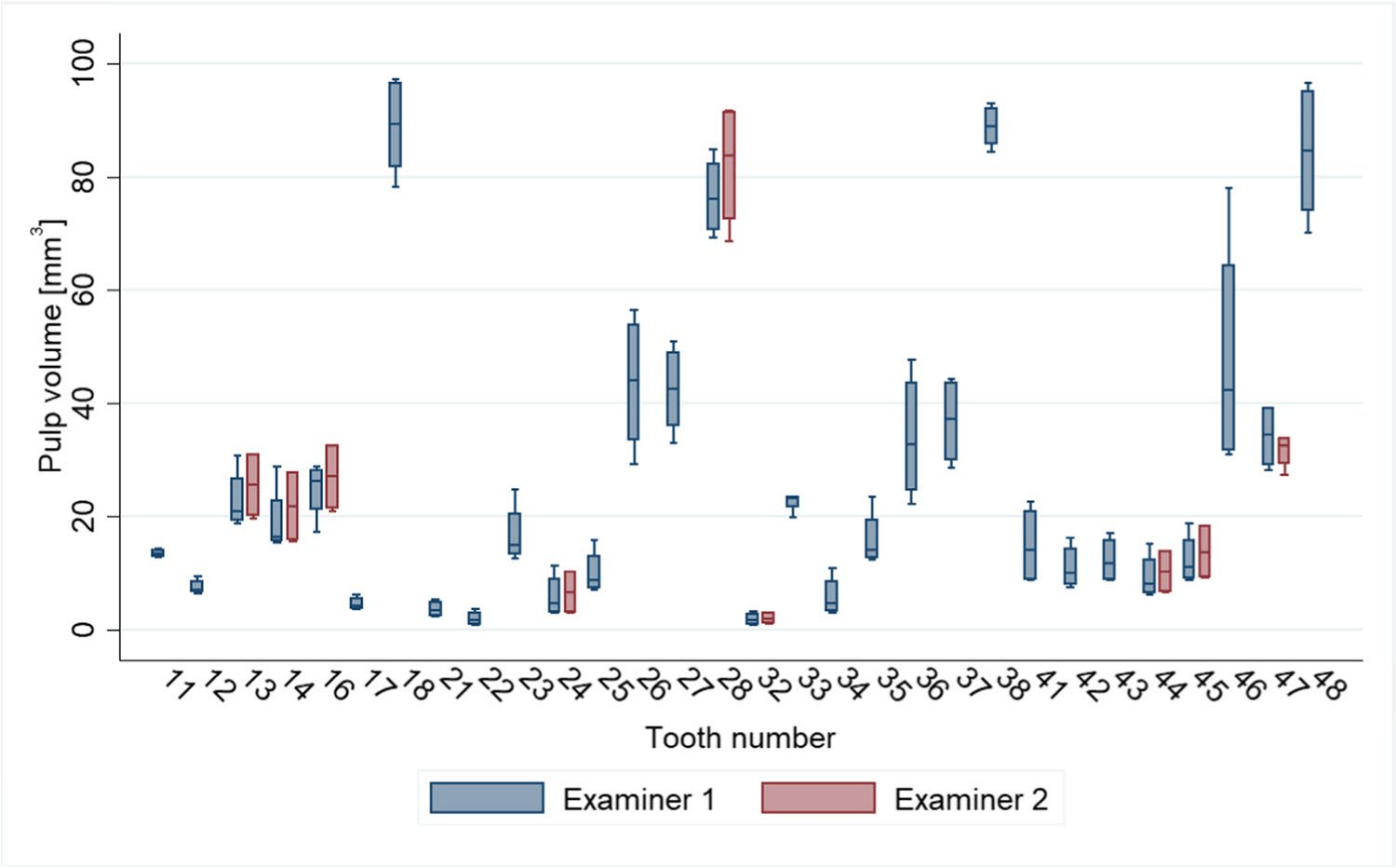
Box plots of each tooth’s pulp volume (mm^3^) for each examiner

Among the nine teeth evaluated by both examiners, the mean pulp volumes from each examiner were similar with overlapping measurements, as inferred from the standard deviations (SDs), although teeth 13, 14, and 16 also had similar, overlapping observations. Plots of the measurements across all teeth show the teeth to be clustered by tooth rather than by examiner (Fig. 2), although there seemed to be greater variation for some teeth, notably tooth 46, which was measured only by examiner 1. Standard deviations across methods and settings ranged from 0.75 (tooth 11) to 20.1 (tooth 46) and from 1.2 (tooth 32) to 11.4 (tooth 28) for examiner 1 and examiner 2, respectively. The ICC measuring absolute agreement was consistently high (ICC > 0.995), indicating good agreement between the two examiners in the measurement of teeth pulp volumes within each combination of method and method setting (Table 1).

**Table 1:** Absolute ICCs measuring agreement between examiners for each configuration of scan method and settings

Rater variable	ICC (95% CI)	Scan method	Scan setting
Examiner	0.997 (0.989, 0.999)	MRI	Semi-automatic
	1.000 (1.000, 1.000)	MRI	Manual
	0.999 (0.991, 1.000)	CBCT	Manual
	0.997 (0.989, 0.999)	CBCT	Semi-automatic

A mixed-effects model was fitted to the 108 observations on teeth common to both examiners, with tooth as a random effect and examiner as fixed, and an interaction between both. While there were significant differences in the levels of tooth, no such differences were detected between the levels of the interaction effect. Fitting an ANOVA model for an omnibus test of the interaction between tooth and examiner as fixed effects did not detect a statistically significant effect, except for the main effect of tooth (*p* value < 0.0001).

### Method agreement

The mean pulp volume determined by CBCT methods was consistently higher with greater variability, as described by the SDs, than that estimated by the MRI method (Table 2). Overall, lower mean pulp volumes had smaller SDs, which may partly have been an artifact of being lower bounded by zero. Examining pulp volumes of only those teeth scanned by both observers, hence affording more observations, it is clear that the variability in measurements from the MRI methods is much smaller than those measured by CBCT, except for teeth 16, 18, 26, and 37.

**Table 2:** Mean pulp volume (SD) by each for both methods (MRI vs. CBCT). The results of the different settings are averaged to two total methods

Tooth	MRI Pulp volume (mm^3^)	CBCT Pulp volume (mm^3^)
11	12.84 (0.11)	14.18 (0.29)
12	6.53 (0.07)	8.52 (0.98)
13	19.49 (0.60)	28.08 (4.29)
14	15.71 (0.21)	24.54 (5.92)
16	21.20 (2.36)	29.73 (2.22)
17	3.64 (0.04)	5.58 (0.70)
18	81.07 (1.91)	96.50 (0.66)
21	7.91 (8.57)	12.36 (11.42)
22	0.92 (0.04)	3.09 (0.74)
23	13.25 (0.45)	20.60 (4.89)
24	3.04 (0.07)	9.35 (2.28)
25	7.21 (0.23)	12.98 (3.21)
26	33.13 (3.63)	53.85 (2.97)
27	35.79 (2.18)	48.87 (2.27)
28	71.26 (2.84)	85.38 (5.24)
32	0.99 (0.02)	2.92 (0.40)
33	23.29 (0.09)	21.71 (2.17)
34	3.18 (0.15)	8.51 (2.79)
35	12.62 (0.18)	19.50 (4.58)
36	24.31 (1.51)	43.50 (4.74)
37	30.08 (1.39)	43.55 (0.89)
38	85.66 (1.44)	92.06 (1.01)
41	8.89 (0.18)	20.86 (2.12)
42	7.86 (0.23)	14.29 (2.29)
43	8.83 (0.12)	15.84 (1.41)
44	6.57 (0.22)	12.90 (2.65)
45	9.11 (0.18)	16.62 (2.95)
46	32.08 (1.33)	64.42 (15.61)
47	29.12 (1.37)	37.34 (2.83)
48	73.36 (2.35)	95.10 (1.76)

In the omnibus (ANOVA) test of effects, fitting the interaction and main fixed effects of tooth and scan method, all terms were highly statistically significant (*p* < 0.0001), indicating not only differences between teeth and scan method, but also differences in how the scan methods measure teeth. Re-fitting as a mixed-effects model with tooth as random, the pulp volumes were on average ~ 43% larger using CBCT than MRI. However, compared to tooth 13 and MRI (the reference levels for the factors in the model), the pulp volumes of teeth 24, 32, 44, and, to a lesser extent, 45 were significantly higher when measured by CBCT.

Absolute agreement between scan methods was still high but this was markedly below those ICCs measuring agreement between the two observers. The ICCs for scan method ranged from 0.886 to 0.910 depending on the stratum of examiner and scan setting (semi-automatic vs. manual) with the lower ICCs for scan methods coming from examiner 1 (Table 3).

**Table 3:** Absolute ICCs measuring agreement between scan methods for each configuration of examiner and scan settings

Rater variable	ICC (95% CI)	Examiner	Scan setting
Scan method	0.889 (-0.124, 0.981)	1	Semi-automatic
	0.903 (-0.117, 0.984)	2	Semi-automatic
	0.886 (-0.140, 0.981)	1	Manual
	0.910 (-0.067, 0.984)	2	Manual

### Setting agreement

The ICCs for scan setting were all very high and did not noticeably vary according to scan method or examiner (Table 4).

**Table 4:** Absolute ICCs measuring agreement between scan settings for each configuration of scan method and examiner

Rater variable	ICC (95% CI)	Scan method	Examiner
Scan setting	0.999 (0.996, 1.000)	MRI	1
	1.000 (1.000, 1.000)	CBCT	1
	1.000 (0.993, 1.000)	MRI	2
	1.000 (1.000, 1.000)	CBCT	2

Since a moderating effect of scan setting might be expected on the method of scanning, then this was explored through backwards elimination from a four-way interaction ANOVA model. However, no significant effect between the scan method and scan setting was found, although the interaction between tooth and scan method was significant (*p* < 0.0001) and the interaction between scan method and examiner, marginally significant (*p* = 0.06).

Further exploration, stratified by scan method, of the interactions between tooth, scan setting, and examiner found that the interaction between tooth and scan setting and the interaction between scan setting and examiner were significant, with *p* values 0.016 and 0.009, respectively, for the MRI method. However, for the CBCT method, no interactions were found to be significant.

### Pulp measurements by root type (single- vs. multi-rooted teeth)

In the ANOVA omnibus test of the backwards elimination from a four-way interaction between method, setting, examiner, and root type, there were significant differences in pulp volume by root type (multi or single), but this did not vary significantly according to whether the scan method was CBCT or MRI, or whether the scan setting was semi-automatic or manual. There was also significant main effect of scan method. However, in fitting a mixed-effects model to account for two-level clustering by root type and tooth, the effect of scan method was significant (*p* < 0.0001), while root type was only weakly significant (*p* = 0.09). From this model, CBCT was on average 73% significantly greater, while multi-root teeth were on average 183% greater, although the latter was estimated with more variation than the former, and so was not significant at the 5% level.

## Discussion

This study compared MRI imaging of teeth to that of CBCT, and expands upon previous work that showed that dental pulp can in principle be imaged by 9.4-T UTE-MRI with a degree of resolution that could be used in legal age assessment [40]. Particular attention should be paid to systematic variations in results between MRI and CBCT and the reproducibility of the two methods. Furthermore, it should be verified whether a conventional software-based segmentation can handle the datasets of MRI and CBCT to the same extent.

The in vitro approach of the present study was performed to examine the teeth without additional noise from other tissues, in order to properly compare the two measurement systems. The work investigates the possibility of switching from a method that involves radiation exposure to a method that does not. Especially in view of the fact that CBCT is associated with higher radiation exposure compared to conventional dental imaging [43–45], the approach of the present study seems to be very reasonable. Thus, the study follows the trend to establish MRI as a radiation-free imaging modality in age assessment, which also includes dental imaging [46–48]. For example, studies are available to assess the mineralization of the third molars using MRI [49]. MRI imaging is expected to become more prevalent in age estimation procedures in the future.

Two teeth showed evidence of a cavity due to carious lesions on imaging and was particularly evident in the MRI imaging. The lesions had not previously been noticed during the inspection before extraction, which has been performed for a different medical indication. The ability to detect caries is essential for forensic imaging methods, as diseased teeth should not be used for age assessment. In this case, there would be a high risk that the tooth would have formed tertiary dentin as a defense reaction, which would not allow a reliable statement about the degree of secondary dentin formation. The present results are consistent with the findings reported in the literature that UTE-MRI imaging is capable of detecting carious lesions [50–52]. Thus, UTE-MRI technology fulfills an important requirement for practical use in forensic age assessment.

**Fig. 3:**
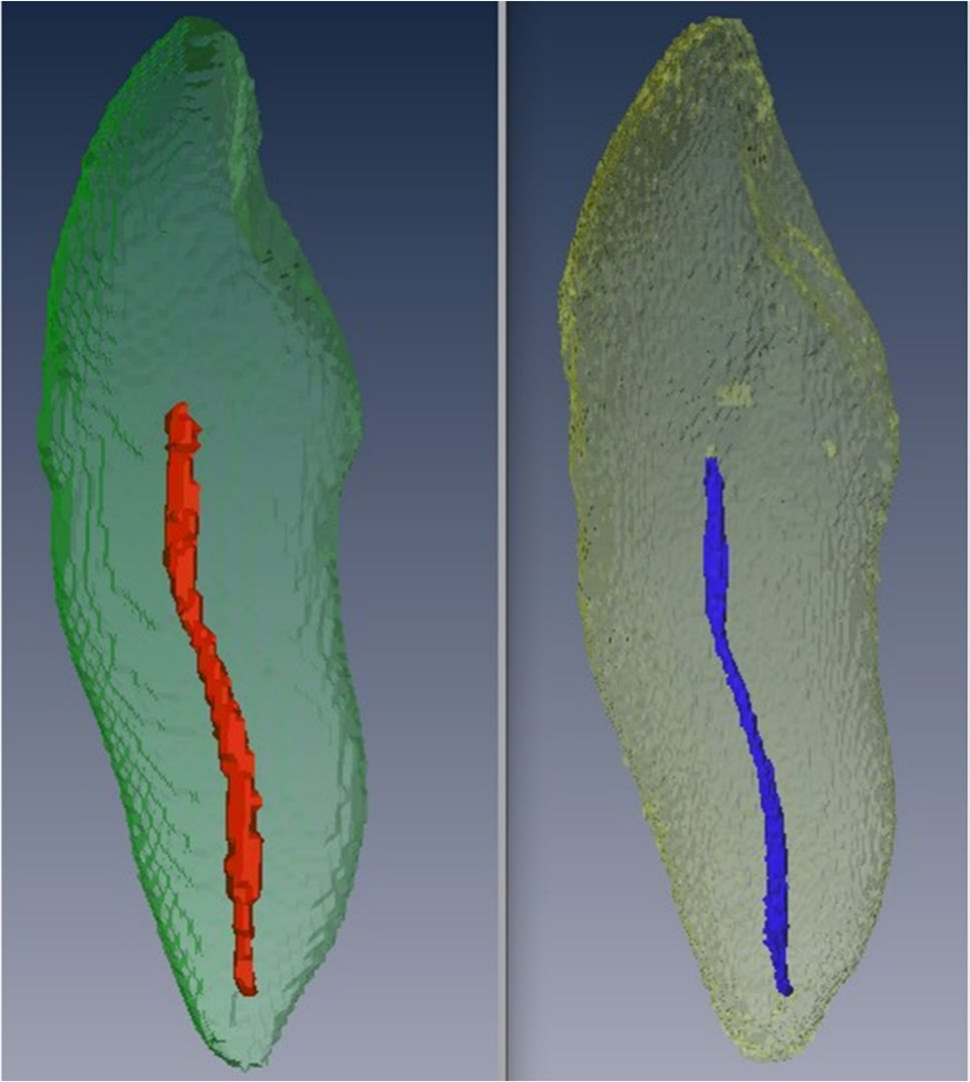
Tooth 11, 3D reconstruction, distal view. Left (red pulp): CBCT dataset, semi-automatic segmentation. Right (blue pulp): MRI dataset, semi-automatic segmentation. The impression of a larger pulp cavity reconstruction in CBCT is already visually striking

**Fig. 4:**
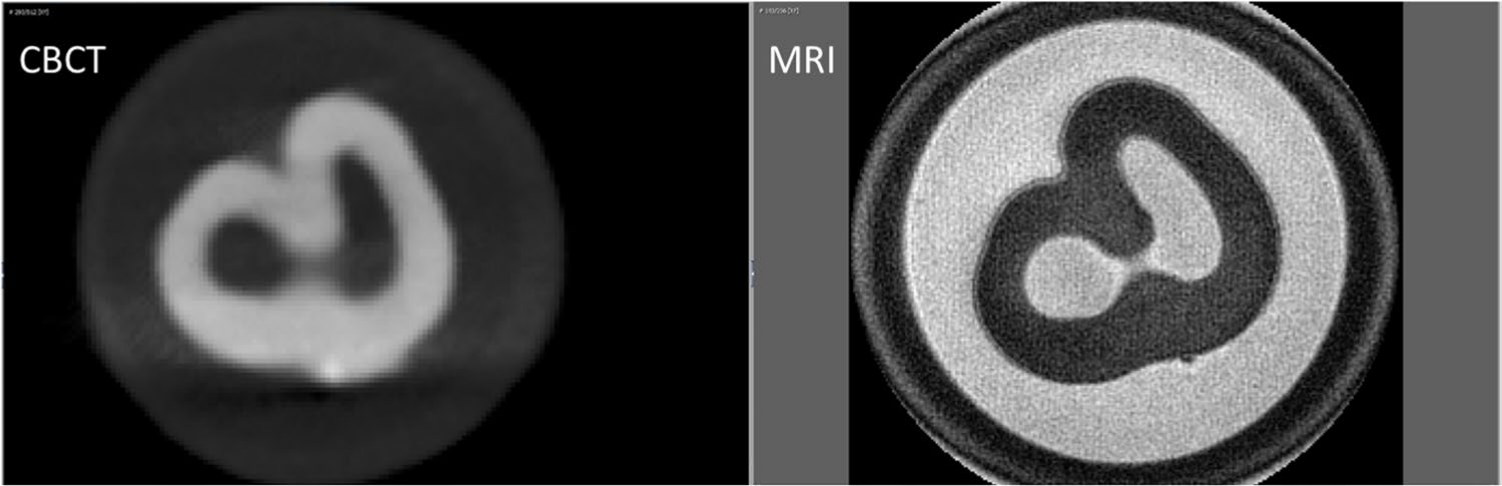
Tooth 48, tooth of 18-year-old male, transversal view. Left, CBCT imaging. Right, MRI imaging. The comparison clearly shows the better sharpness in the MRI images compared to the CBCT. In younger teeth, no changes were detectable within the dental pup on MRI either

**Fig. 5:**
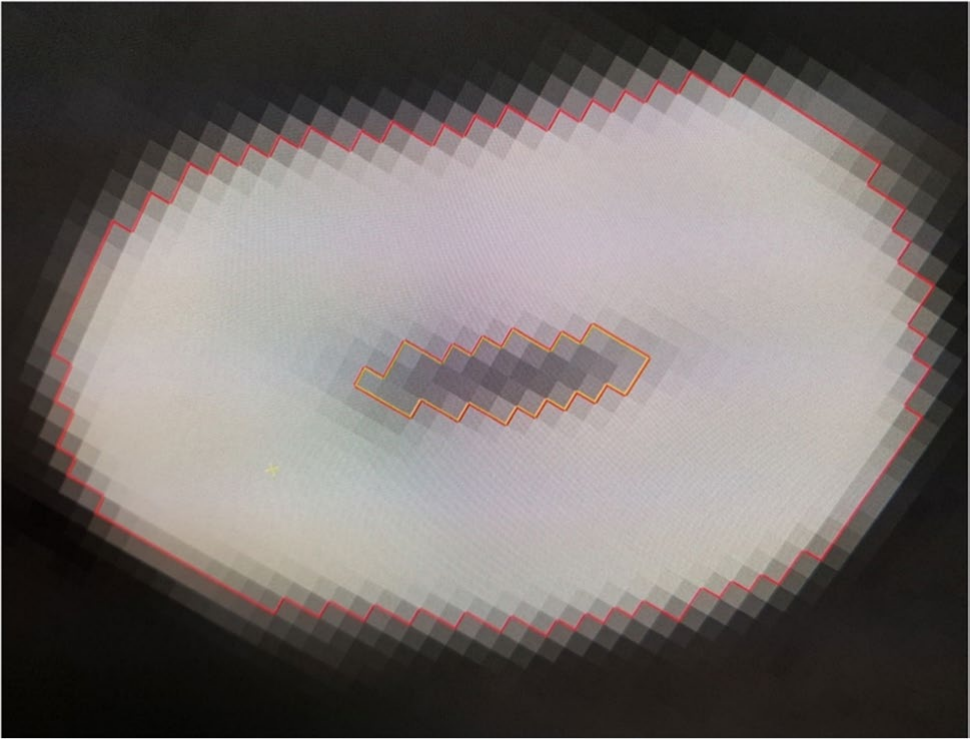
Tooth 43, CBCT dataset, enlarged transversal view. Manual segmentation. Red line, external tooth boundary. Orange line, segmentation of the pulp cavity. Even with semi-automatic segmentation, the examiner must first determine the gray values that are to be considered as pulp by the software

Our results demonstrated reasonable repeatability within the MRI and CBCT methods, and clear differences lay between the two methods. With more variability in the CBCT measurements, the results would seem to favor MRI as a method for pulp cavities. Another very important aspect of the results is that the volume of the pulp was systematically determined to be larger in the CBCT (Fig. 3). Studies are available in the literature that have investigated the actual accuracy of CBCT for measuring the volume of the dental pulp. In 2006, Yang et al. compared the results of determining the pulp volume of 2 teeth using CBCT with the physical measurement of pulp volume using the Archimedes’ principle. The authors found a variation between methods of ± 7.6% [31]. In 2011, Star et al. investigated the pulp volume of 111 teeth using CBCT. They also determined the volume of the pulp physically according to Archimedes’ principle. However, it should be noted that the authors determined the actual pulp volume on only 3 selected teeth of the cohort. The authors found a deviation of the volume of up to 21%. No systematic over- or underestimation is reported [53]. Pinchi et al. (2015) demonstrated a systematic underestimation of pulp volume of 53 to 67% in CBCT compared to a physical measurement based on the examination of 3 teeth [54]. However, it is critical to note here that no anatomical 3D reconstruction of the pulp cavity was performed from the CBCT datasets by Pinchi et al. Rather, Pinchi et al. presented a time-saving approach in which the tooth structures were constructed in the form of simple geometric figures from the 2D CBCT datasets [54]. Thus, this approach is not suitable for stating the actual deviation between the CBCT results and the results of the physical measurements for the pulp volume. Overall, looking at the literature, no systematic measurement error can be derived for CBCT methods. Therefore, there is no evidence that the systematic larger representation of the dental pulp in CBCT compared to MRI is not due to the fact that one method represents the real volumes better than the other. The reported effect is most likely due to the performance of the imaging at the junction between dentin and pulp chamber. Here, the junction in the CBCT is not as sharply drawn, with correspondingly many gray values, as in the MRI, so that borderline gray values were counted to the pulp here. In the MRI datasets, the junction was sharper, so that there was less scope for segmentation (Figs. 4 and 5). This is also reflected in the fact that the variation of results in CBCT was greater than that in MRI. Therefore, Marroquin et al. (2016) already argued in an article on the effect of different methods for calculating the dental pulp that volume measurements with any segmentation method must be understood as an approximation of the measured structure and not as the real volume [39]. The systematic discrepancy between CBCT and MRI demonstrated in the present study indicates that specific reference values or regression formulas for age assessment must be determined for MRI. The application of values determined from CBCT data to MRI datasets could lead to an overestimation of the age and thus to disadvantages of the examined person.

In this study, manual segmentation was compared with semi-automatic segmentation. The approach is considered semi-automatic because the examiner first has to select in one section of the dataset which gray values are to be included in the respective structure [38]. Thus, this approach is also based on a subjective assessment by the examiner. The transversal view of a CBCT image (Fig. 5) illustrates how even a few gray values could have a considerable effect on the calculated volume. The advantage of the semi-automatic setting is that it works through currently available commercial software and it is not necessary to program custom applications first.

**Fig. 6:**
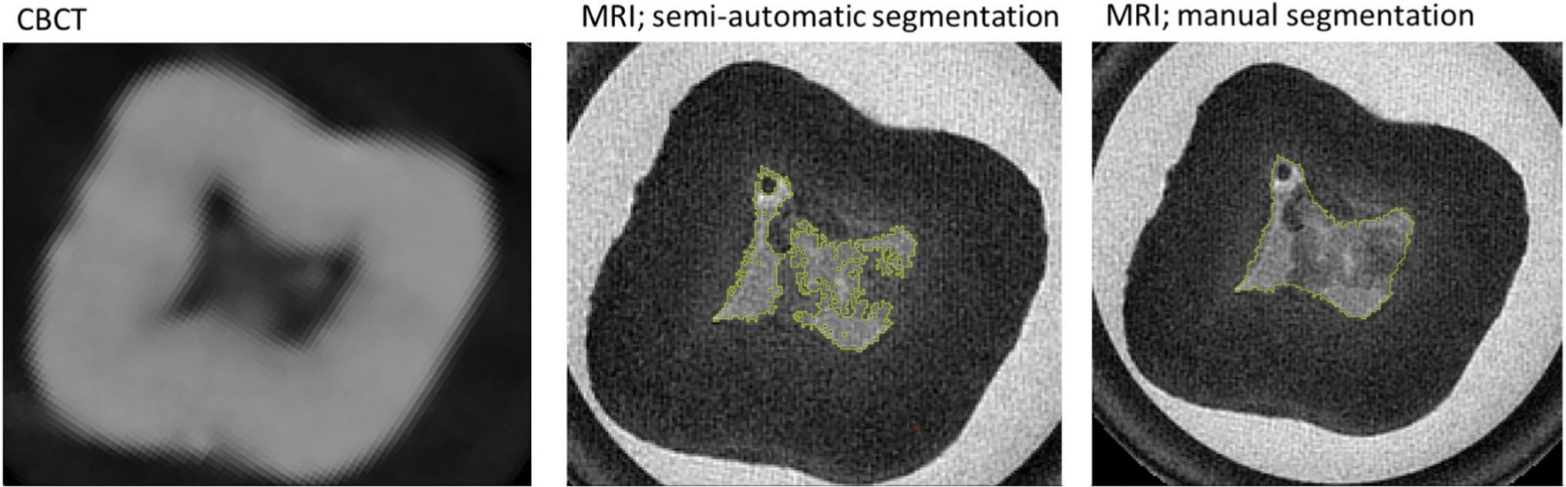
Tooth 37. Tooth of 54-year-old male; transversal view. (“smoothing”; none). Left: CBCT-method. Middle: MRI-method, semi-automatic-setting (yellow line). Right: MRI-method, manual-setting (yellow line). The higher information content about the interior of the dental pulp in the MRI, represented by substantial differences in intensity, leads to a representation of the pulp in the semi-automatic segmentation that cannot be reconciled with the anatomical conditions

In the present study, significant differences were found between manual segmentation and semi-automatic segmentation for MRI. This was not detected for the CBCT datasets, consistent with the results of Marroquin et al. from 2016, who also could not demonstrate any influence of segmentation for CBCT datasets [39]. The effect is most likely due to the fact that the information content about the interior of the dental pulp is higher in MRI (Fig. 6). While the tooth pulp is shown as a largely homogeneous radiolucency with little variation in gray values in the CBCT, different intensities are detected within the pulp in the MRI. According to the impression of the examiners, the amount of these areas increases with age. However, this impression was not statistically examined in the current study. It can be assumed that these intensity changes are caused by degenerative changes of the pulp, as they can also lead, for example, to the formation of denticles [55]. These structures within the high intensities of the dental pulp are incorrectly added to the tooth hard tissue due to their low intensity, in the semi-automatic segmentation, whereas they can be assigned to the dental pulp in the manual procedure based on the examiner’s anatomical knowledge and synopsis with the other slices (Fig. 6). These intensity changes within the pulp on MRI had already been reported by Timme et al. in their 2020 article [40]. In the future, it could be clarified whether the detailed representation of the interior of the dental pulp, on the one hand, can also be detected in vivo and whether this could possibly even be used for age assessment. This detailed manual segmentation may also explain the significant interactions for teeth and setting and for examiner and setting using MRI method, which we detected in the present study. The fact that MRI requires manual segmentation can be considered a disadvantage of the method, although this is due to a higher information content in MRI. For the future, it is desirable that also the more complex representation of the dental pulp in MRI is correctly segmented by software.

Approaches for a full-automatic handling of MRI datasets are already available. Recently, in 2021, Auf der Mauer et al. published a methodology using deep learning to automatically evaluate 3D datasets of the knee for age assessment [56]. However, it is important to keep in mind that convolutional neural networks (CNNs) require training to function optimally. For this process, the relevant structures are usually also segmented by an examiner. Thus, even these methods are partially subjective. In contrast, other authors argue that deep neural networks for image recognition, including CNN, can assume minimally processed input and find optimal network configuration through a self-training procedure [57]. Even with this approach postulated by Lee and Kim in 2018, however, at least manual definition of feature points in the radiograph is necessary. Kim and Lee developed a method for automatic age estimation from hand radiographs using deep learning [57]. However, subjectivity is not completely removed from this approach. On the other hand, results are already available where deep convolutional neural networks (DCNN) were applied to entirely unprocessed imaging datasets for age estimation on MRI datasets of the hand [58]. However, these approaches are currently still error prone or require huge datasets for learning, since the software would then have to detect, for example, anatomical norm variants completely independently [58]. Here, the manifold possible configurations of the dental pulp would make a fully automatic segmentation very challenging. In conclusion, the future points the way to fully automated and examiner-independent imaging evaluation. However, further research is necessary until this goal is reached, especially for complex dental anatomy.

In the present study, complete permanent dentition with 32 teeth (teeth 11–48 [FDI scheme]) was investigated. It was shown that UTE-MRI imaging is basically able to determine the pulp volume for all human teeth or tooth types. Furthermore, we found the measurement of pulp volume was not affected by whether the tooth was single-rooted or multi-rooted. In the literature on the subject, the majority of studies have used single-rooted teeth for age estimation by CBCT imaging. In particular, the canines were examined because it was assumed that these teeth have larger pulp dimensions, subject to less wear from diet and demonstrate high level of survival compared with other teeth in dentition [31, 32, 59–61]. But other anterior teeth were also examined. Studies found that the secondary dentition of the central maxillary incisor correlated better with age than that of the canines [62]. Ge et al. (2016) investigated the question of whether a tooth or tooth type is particularly suitable for age diagnosis using secondary dentin formation [33]. They found that secondary dentin formation of maxillary second molars showed the best correlation with age. However, the authors examined only the crown pulp without considering the root pulp. Ge et al. concluded by stating that multiple types of tooth may improve the accuracy of age estimation compared with only one type of tooth used [35]. Further research is needed in this area. Nevertheless, the MRI method investigated in the present study does not impose any restrictions on tooth types. Thus, the method is basically suitable for future studies in this field.

The in vitro approach does not allow for the visualization of the living, perfused pulp, which is to be considered a shortcoming of the study. Pulp vitality could have an influence on MRI imaging, although an influence on CBCT imaging cannot be assumed. On the other hand, the approach as presented in this paper is just as applicable, e.g., for the age assessment of unknown dead bodies. In addition, the fact that a correlation of pulp volume and age was not performed is also considered a shortcoming of the study. However, it was not the purpose of the study to establish reference values, and the basic suitability of assessing secondary dentin formation for age estimation can be regarded as proven.

## Conclusions

For visualizing the pulp volume of teeth, 9.4-T UTE-MRI is a reliable method. Currently, unlike CBCT, manual segmentation is required for the MRI datasets due to the more complex visualization of the pulp chamber. Equally suitable is 9.4-T UTE-MRI for imaging both single-rooted and multi-rooted teeth. The MRI approach seems to be more prone to image artifacts than the CBCT approach, at least in the in vitro setting. Compared to CBCT, the variation of the measurement results is smaller, with the pulp volume being systematically displayed smaller in MRI. For this reason, method-specific reference values are mandatory. The results of the present study should be verified in future in vivo studies.

## Declarations

Ethical approval for the study was obtained from the Ethics Committee of the Medical Association of Westfalen-Lippe and of the University of Muenster, Münster, Germany (2017–215-f-S).
